# Two-Phase Orthodontic Treatment in an Adolescent With a 15 mm Severe Overjet

**DOI:** 10.7759/cureus.99080

**Published:** 2025-12-12

**Authors:** Shuhao Xu, Xiaolong Li, Yu Zhang, Wei Li

**Affiliations:** 1 Department of Stomatology, Deyang People's Hospital, Deyang, CHN; 2 Department of Pulmonary and Critical Care Medicine, Deyang People's Hospital, Deyang, CHN

**Keywords:** adolescent, case report, class ii malocclusion, maxillary expansion, two-phase orthodontic treatment

## Abstract

Angle Class II division 1 malocclusion is a type of malocclusion that significantly impacts the psychological health of adolescents. The key to treating Angle Class II division 1 malocclusion lies in targeting the peak pubertal growth period and fully utilizing the mandible's growth potential. We present the case of a 10-year-old boy diagnosed with Angle Class II division 1 malocclusion, characterized by a severe overjet of 15 mm, managed through a two-phase orthodontic treatment. Phase I of the treatment involved maxillary expansion to eliminate mandibular growth restrictions and retract the proclined anterior teeth. Phase II comprised comprehensive orthodontics with the extraction of four premolars to achieve further anterior retraction, improve the convex soft tissue profile, and establish a harmonious occlusion. After 25 months of this two-phase treatment, significant improvement in the soft tissue profile was observed, with effective anterior retraction, well-aligned dentition, and functional occlusion.

## Introduction

Angle Class II division 1 malocclusion is characterized by proclined upper anterior teeth, distal molar relationship, and deep overbite and overjet. It is a type of malocclusion that significantly impacts the psychological health of adolescents [1]. Previous studies have shown that up to approximately one in three individuals is affected by Class II malocclusion [1,2]. The severity of malocclusion may be related to disease progression and can potentially worsen with age [3]. Beyond aesthetic concerns and psychological impact, malocclusion can also affect oral functions such as chewing and swallowing, lead to occlusal trauma, and increase the risk of dental injury [4]. Therefore, implementing effective treatment strategies for Angle Class II division 1 malocclusion is crucial to halt its progression [5].

The treatment options for Angle Class II division 1 malocclusion primarily include functional appliances, fixed camouflage orthodontics, clear aligner therapy, and combined orthodontic-orthognathic surgery. Comparative randomized data on aligners versus fixed appliances further inform appliance selection [6]. It is currently believed that the key to treating Angle Class II division 1 malocclusion lies in targeting the peak pubertal growth period and fully utilizing the mandible's growth potential. The optimal treatment window is considered to be one year before the onset of this peak growth phase [7]. Randomized trials have documented modest dentoalveolar and soft-tissue changes with functional appliances in growing Class II patients [8]. However, for Angle Class II division 1 malocclusion accompanied by deep overbite causing occlusal trauma, proclined anterior teeth increasing the risk of dental injury, detrimental oral habits, or the presence of factors interfering with mandibular advancement, such as maxillary dental arch constriction or individual anterior tooth lingual version, early orthodontic intervention should be initiated to eliminate these interfering factors and remove restrictions on forward mandibular growth [9].

A two-phase orthodontic treatment (functional appliance therapy followed by fixed appliances) is being increasingly employed to improve both facial profile and occlusal relationship in patients with Angle Class II division 1 malocclusion. For patients with Angle Class II division 1 malocclusion presenting with crowding and/or pronounced proclination, a Phase I functional appliance therapy is often recommended, typically followed by premolar extractions to alleviate crowding and/or improve the facial profile [10].

This article presents a case report on the orthodontic management of an adolescent with Angle Class II division 1 malocclusion, characterized by a severe overjet of 15 mm. In this case, in addition to severe proclination of the upper anterior teeth and lip incompetence, which posed a significant risk of dental trauma, constriction of the maxillary dental arch was also present. Therefore, a two-phase orthodontic treatment plan was implemented. Phase I involved maxillary arch expansion to coordinate the dental arches, retraction of the proclined anterior teeth, and elimination of restrictions on mandibular growth. Phase II comprised comprehensive orthodontic treatment with extraction of four premolars to achieve further anterior retraction, improvement of the convex soft tissue profile, and establishment of a harmonious occlusion. The two-phase orthodontic treatment was completed over 25 months, resulting in significant improvement of the soft tissue profile, effective anterior retraction, well-aligned dentition, and functional occlusion, achieving satisfactory therapeutic outcomes.

## Case presentation

A 10-year-old boy presented to our department with a chief complaint of severely proclined newly erupted anterior teeth observed for over two years. His medical history was unremarkable except for adenoid and tonsillectomy, with no other systemic diseases or drug allergies reported. The parent reported that the boy had a history of mouth breathing, which had resolved following surgery.

**Figure 1:**
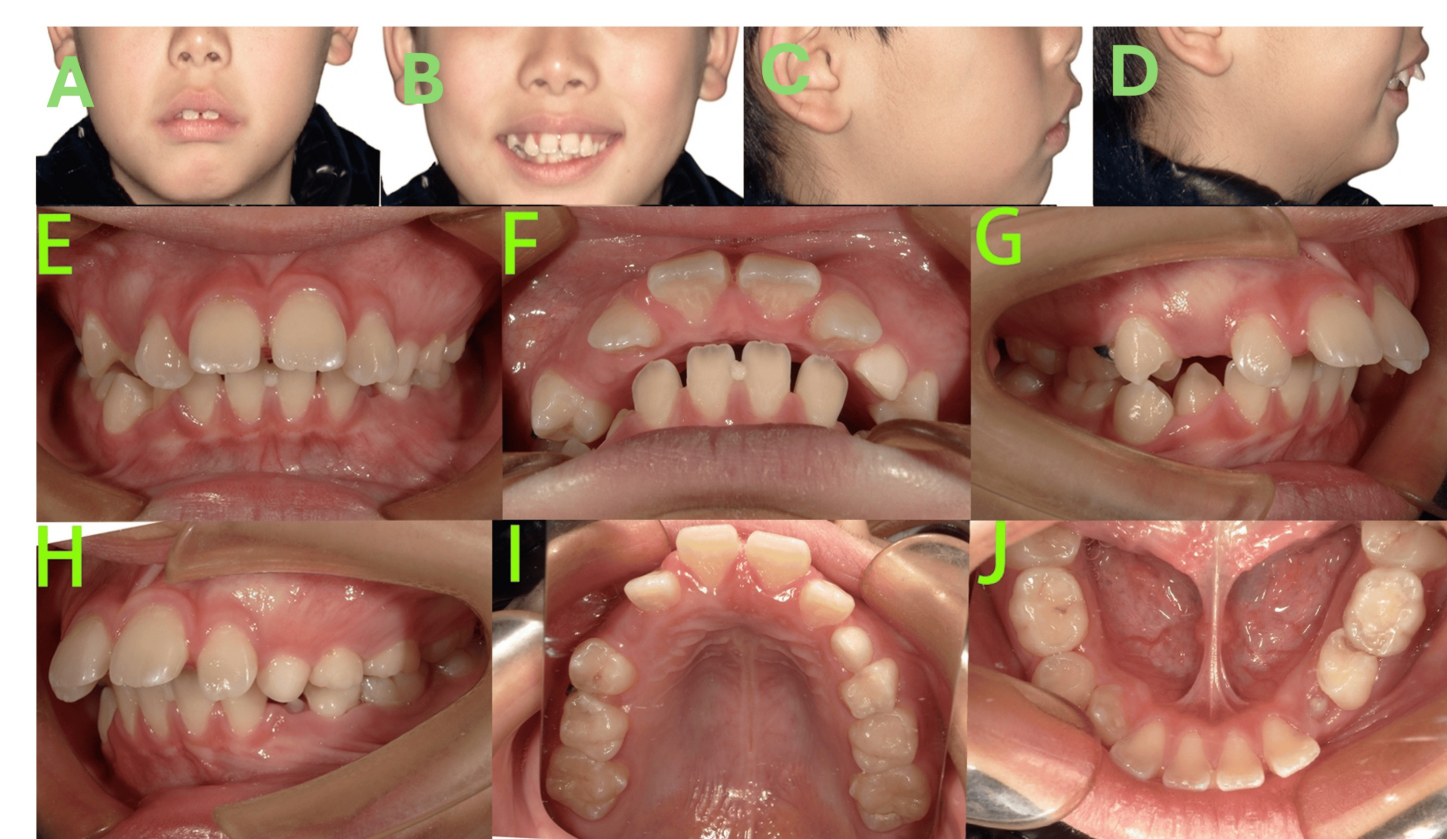
Pre-treatment photos A-D: Facial photos revealed a convex profile, mandibular retrognathia, increased mandibular angle, shallow mentolabial sulcus, mentalis muscle strain, acute nasolabial angle, mesocephalic facial type, and lip incompetence. E-J: Intraoral photos revealed the patient was in the mixed dentition stage, bilateral molar relationships were full Class II, the maxillary arch was constricted, the upper anterior teeth were severely proclined, exhibiting a 15 mm overjet and a Grade III deep overbite (palatal impingement), and carious lesions were noted on teeth 64, 65 and 74.

Facial examination (Figures 1A-1D) revealed a convex profile, mandibular retrognathia, increased mandibular angle, shallow mentolabial sulcus, mentalis muscle strain, acute nasolabial angle, mesocephalic facial type, and lip incompetence. At rest, the lower lip was positioned lingual to the upper incisors.

Intraoral examination (Figures 1E-1J) revealed the following findings: The patient was in the mixed dentition stage with lateral tooth groups undergoing eruption, specifically teeth 16, 14, 12-22, 26, 36, 33-44, and 46 had already erupted. Bilateral molar relationships were full Class II. Diastemas were present in both upper and lower anterior regions. The maxillary arch was constricted with a tapered form, contrasting with the ovoid shape of the mandibular arch, resulting in significant arch discrepancy. The upper anterior teeth were severely proclined, exhibiting a 15 mm overjet and a Grade III deep overbite (palatal impingement). Dental midlines were essentially normal. Carious lesions were noted on the distal-occlusal surface of tooth 64, mesial-occlusal surface of tooth 65, and distal-occlusal surface of tooth 74.

**Figure 2:**
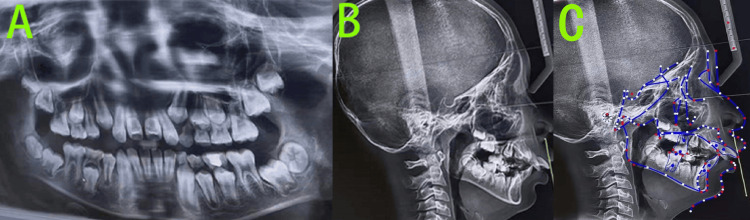
Pre-treatment radiographs A: Panoramic radiograph revealed the number of permanent tooth buds was within normal limits; B: Lateral cephalogram; C: Cephalometric tracing indicated a skeletal Class II pattern with mandibular retrognathia, a high mandibular plane angle, an average growth pattern, severely proclined upper incisors, and labially inclined lower incisors. Cephalometric tracing in Figure 2C created using the UCeph software (version 3.1, UCeph Software, Chengdu, China).

The pre-treatment panoramic radiograph (Figure 2A), obtained from an external institution, revealed the following: the number of permanent tooth buds was within normal limits.

Root resorption was evident in teeth 64 and 65, while tooth 74 exhibited complete root resorption.

We performed a cephalometric analysis (Table 1) on the pre-treatment lateral cephalogram (Figure 2B) taken at an external institution.

**Table 1:** Pre-treatment cephalometric measurements SNA: Sella-Nasion-A; SNB: Sella-Nasion-B; ANB: A-Nasion-B; Wits: Wits Appraisal; S-Go/N-Me: Sella-Gonion/Nasion-Menton (the ratio of posterior to anterior lower facial height); FMA: Frankfort-mandibular plane angle; U1-SN: Upper Incisor to Sella-Nasion Plane; IMPA: incisor mandibular plane angle.

Cephalometric parameters	Measured value	Reference value
SNA(°)	79.4	83.0±4.0
SNB(°)	70.6	83.0±4.0
ANB(°)	8.8	3.0±2.0
Wits(mm)	6.3	0.0±2.0
S-Go/N-Me (%)	62.1	64.0±2.0
FMA(°)	31.8	26.0±4.0
U1-SN(°)	130.9	106.0±6.0
IMPA(°)	106.6	97.0±6.0

Cephalometric analysis (Figure 2C) was performed by the same orthodontist using UCeph software (version 3.1, UCeph Software, Chengdu, China), with manual landmark identification followed by automatic software measurement. To minimize error, the measurements were conducted three times, and the average value was taken. Cephalometric analysis showed cervical vertebral maturation stage (CVS) 2, indicating pre-peak growth potential. The skeletal pattern was Class II with mandibular deficiency, accompanied by a high mandibular plane angle and an average growth pattern. The upper incisors were severely proclined, and the lower incisors were also labially inclined.

Diagnosis showed dental caries in teeth 64, 65, and 74. Pre-peak growth potential with the following skeletal and dental characteristics was observed: skeletal Class II pattern with mandibular deficiency, high mandibular plane angle, average growth pattern, severe proclination of upper incisors, labial inclination of lower incisors; constricted maxillary arch; and diastemas in both dental arches.

After critical evaluations, our treatment plan consisted of the following components: oral hygiene instruction; scheduling regular follow-up appointments to monitor the exfoliation of primary teeth and the eruption of permanent dentition; implementing lip sealing exercises to improve perioral muscle tone and competence; and a two-phase orthodontic treatment.

Phase I included utilizing a maxillary slow expansion appliance accompanied by a double-loop lip bumper. The primary objectives were to expand the constricted maxillary arch, coordinate the maxillary and mandibular arch forms, and achieve retraction of the proclined upper anterior teeth. The decision to proceed with mandibular advancement functional appliance therapy was contingent upon the evaluation of intraoral occlusion and subsequent cephalometric analysis results. Phase II consisted of a comprehensive fixed orthodontic treatment. The potential for tooth extractions remained a consideration, pending further evaluation, to address crowding and profile convexity.

The patient's parents were thoroughly informed of the diagnosis and treatment plan, and serial treatment was initiated after obtaining their informed consent. At the start of treatment, we performed maxillary slow expansion using a removable maxillary split baseplate. The expansion screw was activated twice a week, with each activation opening the screw by 0.25 mm.

**Figure 3:**
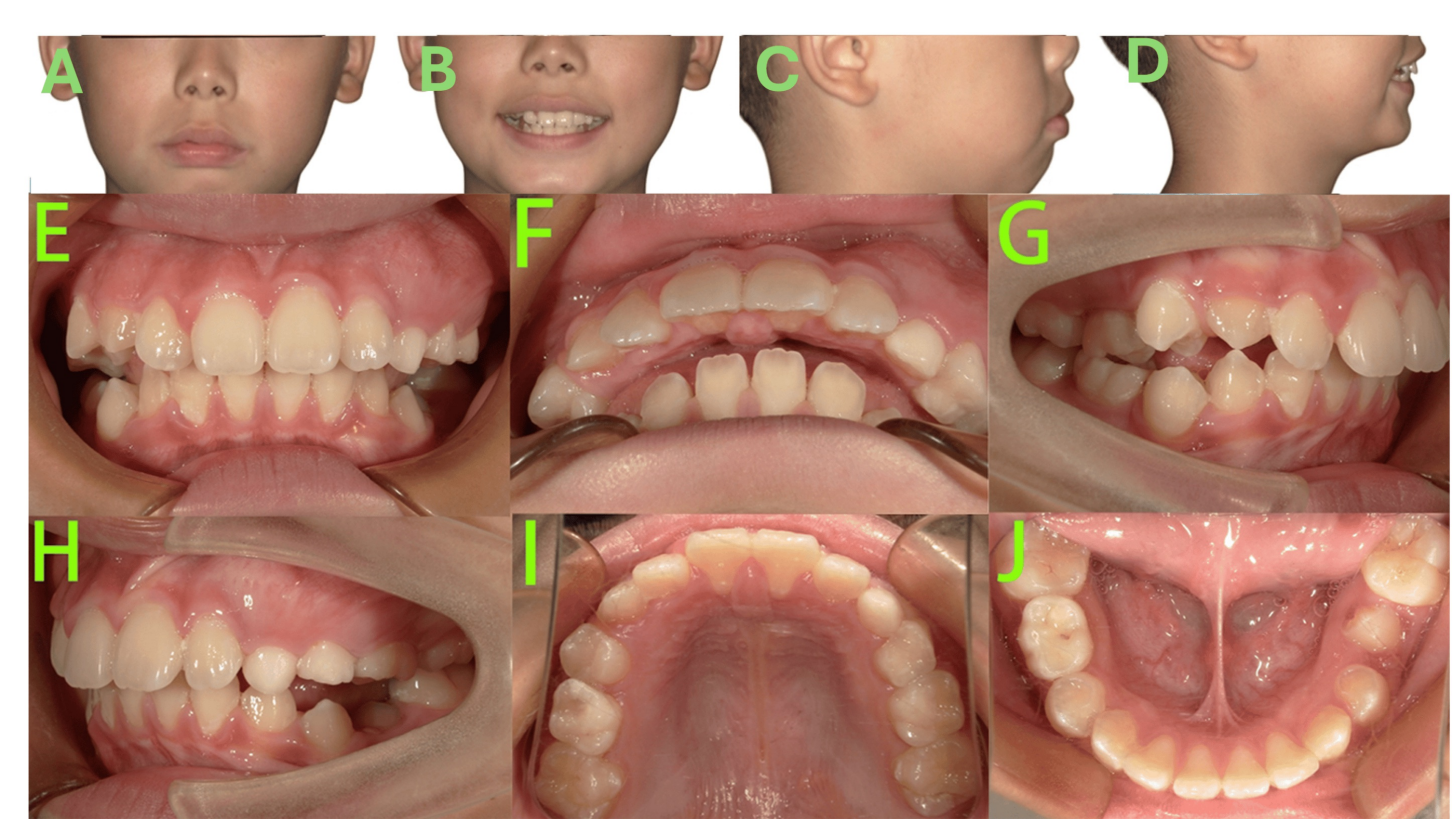
Phase I treatment photos after five months A-D: Facial photos showed lip seal competence was enhanced, and the convex facial profile showed notable improvement. E-J: Intraoral photos showed the upper anterior teeth were retracted, with closure of the diastemas in the upper anterior region, reducing the overjet to 7 mm, establishment of a normal overbite, and adjustment of the bilateral molar relationship to an end-on Class II.

After five months of Phase I maxillary expansion therapy (Figure 3), significant improvements were observed.

Lip seal competence was enhanced, and the convex facial profile showed notable improvement. The maxillary expansion was effective, resulting in coordinated maxillary and mandibular arch forms. The upper anterior teeth were retracted, with closure of diastemas in the upper anterior region, reducing the overjet to 7 mm. The deep overbite was corrected, establishing a normal overbite. The bilateral molar relationship was adjusted to an end-on Class II. The primary teeth 55, 63-65, and 85 remained unexfoliated, and the patient was still in the pre-peak growth stage. The expansion appliance was deactivated but maintained in place for retention. Regular follow-ups were scheduled to monitor the exfoliation of the remaining primary teeth while awaiting the peak pubertal growth period.

**Figure 4:**
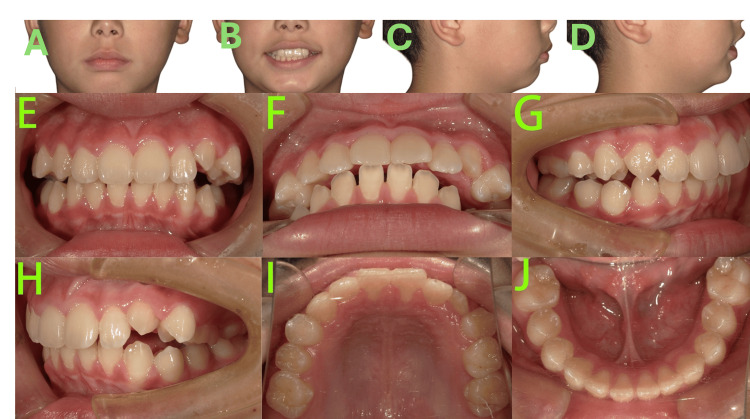
Pre-phase II treatment photos A-D: Facial photos showed competent lip seal was achieved, and the convex facial profile demonstrated marked improvement compared to the initial presentation. E-J: Intraoral photos showed the maxillary and mandibular arch forms were well-coordinated, retraction of the upper anterior teeth had been accomplished, resulting in an overjet reduction to 3 mm and a normalized overbite, and the molar relationship was corrected to Class I on the left side and a mild Class II on the right.

Following six months of retention after Phase I maxillary expansion (Figure 4), significant progress was noted.

Competent lip seal was achieved, and the convex facial profile demonstrated marked improvement compared to the initial presentation. The maxillary and mandibular arch forms were well-coordinated. Retraction of the upper anterior teeth had been accomplished, with closure of the pre-existing diastemas, resulting in an overjet reduction to 3 mm and a normalized overbite. The molar relationship was corrected to Class I on the left side and a mild Class II on the right. The primary tooth 65 remained the only deciduous tooth yet to exfoliate.

**Figure 5:**
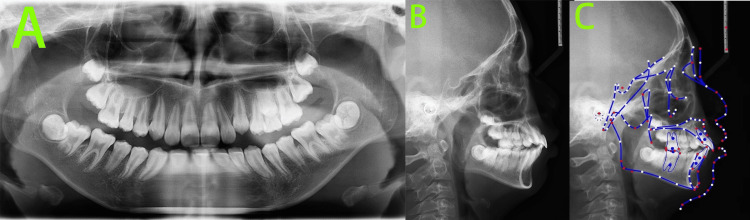
Pre-phase II radiographs A: Panoramic radiograph revealed root resorption of tooth 65; B: Lateral cephalogram; C: Cephalometric tracing indicated significant retraction of the upper anterior teeth and an improvement in the skeletal Class II relationship. Cephalometric tracing in Figure 5C created using the UCeph software (version 3.1, UCeph Software, Chengdu, China).

A follow-up panoramic radiograph (Figure 5A) revealed root resorption of tooth 65. A subsequent lateral cephalogram (Figure 5B) was obtained to facilitate the planning and initiation of comprehensive Phase II fixed orthodontic treatment.

**Figure 6:**
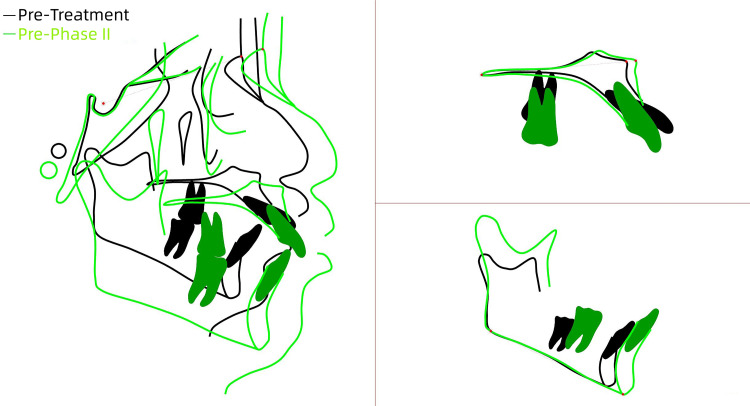
Superimposition of the pre-phase II and pre-treatment tracings The superimposition of the pre-phase II (green line) and pre-treatment (black line) tracings demonstrated significant retraction of the upper anterior teeth, with no apparent forward mandibular rotation observed. Image credits: Created by Xu S using UCeph software (version 3.1, UCeph Software, Chengdu, China).

A comprehensive cephalometric analysis (Figure 5C) was performed and compared with the initial records (Table 2, Figure 6).

**Table 2:** Pre-phase II cephalometric measurements SNA: Sella-Nasion-A; SNB: Sella-Nasion-B; ANB: A-Nasion-B; Wits: Wits Appraisal; S-Go/N-Me: Sella-Gonion/Nasion-Menton (the ratio of posterior to anterior lower facial height); FMA: Frankfort-mandibular plane angle; U1-SN: Upper Incisor to Sella-Nasion Plane; IMPA: incisor mandibular plane angle.

Cephalometric parameters	Pre-treatment	Pre-phase II treatment	Reference value	Changes after phase I treatment
SNA(°)	79.4	79.1	83.0±4.0	-0.3
SNB(°)	70.6	72.2	83.0±4.0	1.6
ANB(°)	8.8	6.9	3.0±2.0	-1.9
Wits(mm)	6.3	5.9	0.0±2.0	-0.4
S-Go/N-Me (%)	62.7	62.5	64.0±2.0	-0.2
FMA(°)	31.8	32.1	26.0±4.0	0.3
U1-SN(°)	130.9	110.2	106.0±6.0	-20.7
IMPA(°)	106.6	105.8	97.0±6.0	-0.8

The analysis indicated CVS 3 stage, confirming the patient's entry into the peak growth period. Significant retraction of the upper anterior teeth was achieved, with some improvement in the skeletal Class II relationship. No significant forward rotation of the mandible was observed.

Given the persistent labial inclination of both upper and lower incisors, the partial self-correction of overjet and molar relationship, and the patient's high-angle vertical pattern, which contraindicates mandibular advancement functional therapy, a decision was made to proceed directly with Phase II extraction-based treatment. The definitive plan involved comprehensive full-mouth fixed orthodontic therapy with the extraction of teeth 65, 14, 24, 35, and 45. The treatment objectives were to align and level both dental arches, achieve further retraction of the anterior teeth, close the extraction spaces, establish Class I molar and canine relationships, achieve normal overbite and overjet, perform detailed occlusal finishing, and provide retention.

**Figure 7:**
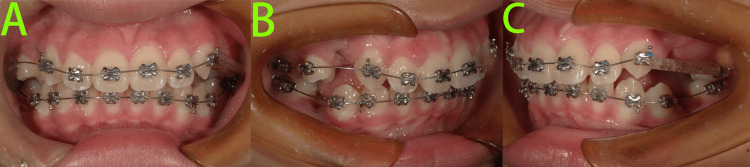
Photos at the commencement of phase II fixed appliance treatment A-C: Intraoral photos showed that following the extraction of teeth 65, 14, 24, 35, and 45, and initiation of the comprehensive fixed orthodontic treatment in Phase II.

Following the extraction of teeth 65, 14, 24, 35, and 45, comprehensive Phase II fixed orthodontic treatment was initiated (Figure 7).

**Figure 8:**
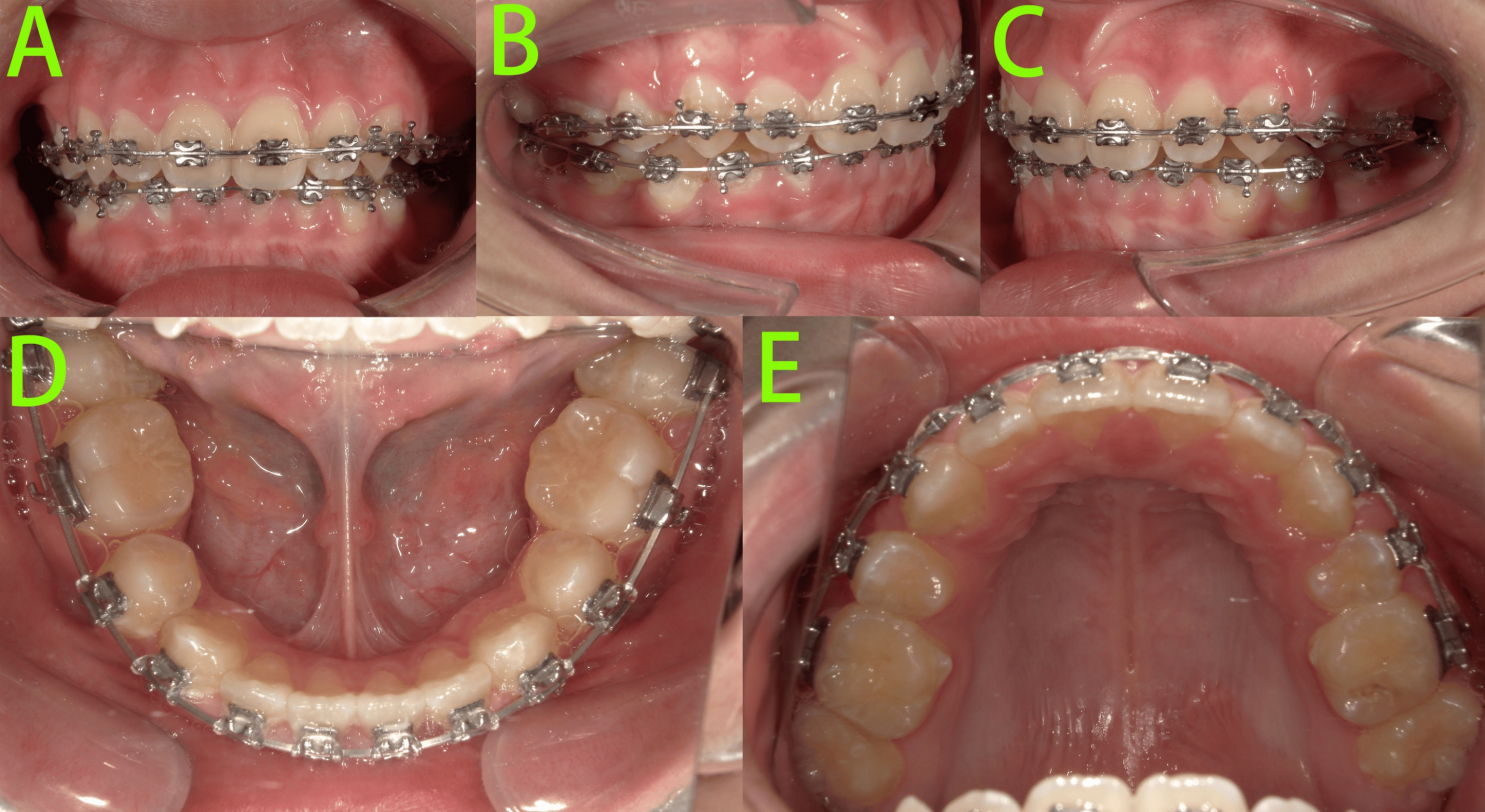
Photos at eight months of Phase II treatment A-E: Intraoral photos show that both dental arches were well-aligned with notable retraction of the upper and lower anterior teeth, and the extraction spaces were largely closed.

Following eight months of Phase II fixed appliance therapy (Figure 8), significant progress was observed. Both dental arches were well-aligned, with notable retraction of the upper and lower anterior teeth. The extraction spaces were largely closed, and the second molars, having erupted, were incorporated into the appliance system.

**Figure 9:**
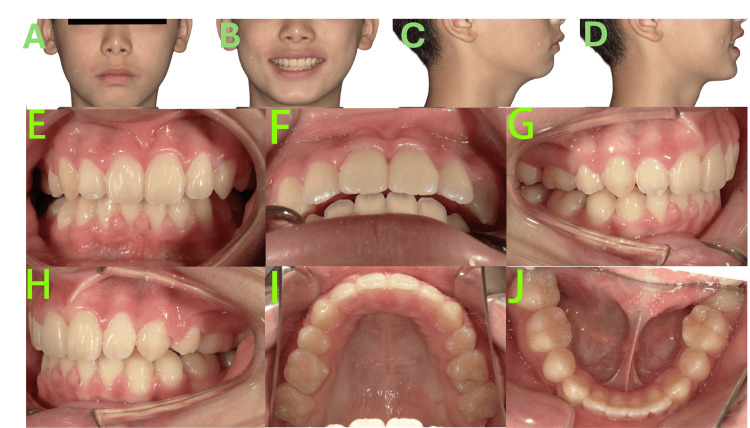
Post-treatment photos after Phase II A-D: Facial photos showed the convexity of the soft tissue profile was significantly improved, resulting in a straight profile, although mandibular retrognathia persisted, and lip seal was achieved naturally. E-J: Intraoral photos showed the maxillary and mandibular dentition were well-aligned and spaced appropriately with bilateral Class I molar and canine relationships, with normal overbite and overjet.

Upon completion of 12 months of Phase II fixed treatment, marking a total treatment duration of 25 months for the combined two-phase therapy (Figure 9), the following outcomes were achieved: the convexity of the soft tissue profile was significantly improved, resulting in a straight profile, although mandibular retrognathia persisted; lip seal was achieved naturally; and the maxillary and mandibular dentition were well-aligned and spaced appropriately.

Bilateral Class I molar and canine relationships were established, with normal overbite and overjet, coincident dental midlines, and firm interdigitation in the lateral segments. The patient and his parents expressed high satisfaction with the treatment outcome. The case then entered the retention phase. The patient was instructed to wear vacuum-formed retainers at daytime and Begg retainers (Pairi Dental Laboratory Co., Chengdu, China) at night. Subsequent follow-up appointments were scheduled at three- to six-month intervals.

**Figure 10:**
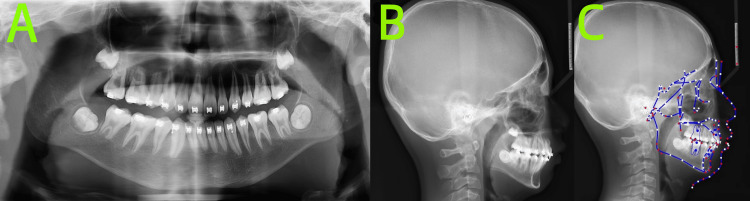
Post-treatment panoramic radiographs after Phase II A: Panoramic radiograph revealed no significant root resorption and good root parallelism throughout the dentition; B: Lateral cephalogram; C: Cephalometric tracing indicated significant retraction of both the upper and lower anterior teeth. Cephalometric tracing in Figure 2C created using the UCeph software (version 3.1, UCeph Software, Chengdu, China).

A final panoramic radiograph obtained prior to debonding (Figure 10) revealed no significant root resorption and good root parallelism throughout the dentition.

**Figure 11:**
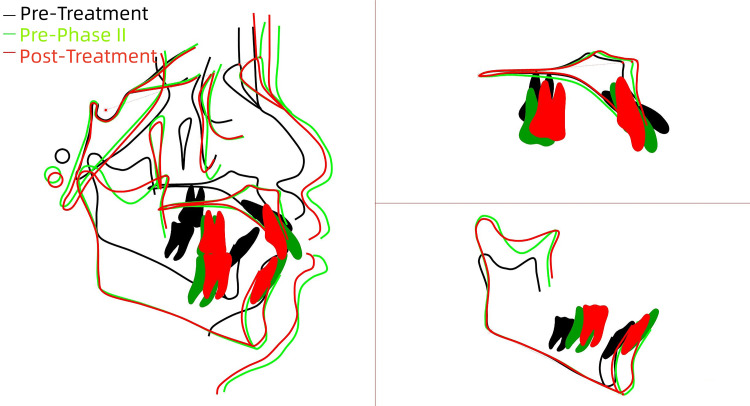
Superimposition of the pre- and post-treatment tracings The superimposition of cephalometric tracings from the three stages—pre-treatment (black line), pre-phase II (green line), and post-treatment (red line)—indicated effective retraction of both the upper and lower anterior teeth, improvement in the skeletal Class II relationship, and no significant clockwise rotation of the mandible. (Image credits: Created by Xu S using UCeph software (version 3.1, UCeph Software, Chengdu, China).

A final cephalometric analysis (Figure 10C) was conducted and compared with earlier records (Table 3 and Figure 11).

**Table 3:** Post-treatment cephalometric measurements after Phase II SNA: Sella-Nasion-A; SNB: Sella-Nasion-B; ANB: A-Nasion-B; Wits: Wits Appraisal; S-Go/N-Me: Sella-Gonion/Nasion-Menton (the ratio of posterior to anterior lower facial height); FMA: Frankfort-mandibular plane angle; U1-SN: Upper Incisor to Sella-Nasion Plane; IMPA: incisor mandibular plane angle.

Cephalometric parameters	Pre-treatment	Pre-phase II treatment	Post-phase II treatment	Reference value	Changes before and after treatment
SNA(°)	79.4	79.1	79.2	83.0±4.0	-0.2
SNB(°)	70.6	72.2	73.3	83.0±4.0	2.7
ANB(°)	8.8	6.9	5.9	3.0±2.0	-2.9
Wits(mm)	6.3	5.9	3.3	0.0±2.0	-3.0
S-Go/N-Me (%)	62.7	62.5	63.0	64.0±2.0	0.3
FMA(°)	31.8	32.1	30.2	26.0±4.0	-1.6
U1-SN(°)	130.9	110.2	90.7	106.0±6.0	-40.2
IMPA(°)	106.6	105.8	100.4	97.0±6.0	-6.2

The analysis demonstrated a significant retraction of both upper and lower anterior teeth, improvement in the skeletal Class II relationship, and an absence of significant forward mandibular rotation.

## Discussion

The etiology of malocclusion is particularly important for the treatment of malocclusion. Etiological treatment must be given the highest priority in determining any treatment plan for malocclusion, because successful treatment is impossible unless the factors causing the deformity are identified and eliminated [11]. The inheritance of malocclusion has the characteristics of polygenic inheritance, meaning that both genetic and environmental factors play a role, with environmental factors acting against a genetic background. Common environmental factors for Class II malocclusion in adolescents include systemic diseases, tooth replacement disorders, functional factors, and oral habits, among which mouth breathing is a common environmental factor [7].

Children who breathe through their mouths often present with "adenoid adenoid faces" [12]. Its characteristics include inadequate upper lip function, retracted hyoid bone, constricted maxillary dental arch, retroclined mandibular incisors, increased anterior facial height, narrow or V-shaped maxillary arch, increased mandibular plane angle, and posterior rotation of the mandible. Mouth breathing is a form of respiration that serves as an alternative to nasal breathing, with complex underlying causes. It may result from genetic factors, detrimental oral habits, or nasal obstruction due to conditions such as, but not limited to, adenoid/tonsillar hypertrophy, nasal polyps, deviated nasal septum, turbinate hypertrophy, or sinusitis [13]. In this case, the patient exhibited a flaccid upper lip, a constricted maxillary dental arch, protrusion of the upper anterior teeth, and a tendency toward clockwise rotation of the mandible. Based on the patient's history of mouth breathing and his medical history of adenoid and tonsillectomy, it was inferred that his Class II malocclusion may be associated with his mouth breathing habit.

The key to treating Class II division 1 malocclusion lies in targeting the peak pubertal growth period and capitalizing on the mandible's inherent growth potential [7]. However, for Angle Class II division 1 malocclusion accompanied by deep overbite causing occlusal trauma, proclined anterior teeth increasing the risk of dental injury, detrimental oral habits, or the presence of factors interfering with mandibular advancement, such as maxillary and maxillary dental arch constriction or individual anterior tooth lingual version, early orthodontic intervention should be initiated. This will eliminate these interfering factors and remove restrictions on forward mandibular growth [9]. For growing patients with a deep bite or functional restrictions, comparative evidence on corrective methods is available [14]. Recent clinical trials on en-masse retraction in Class II division 1 patients provide comparative benchmarks for rate and root outcomes [15]. In this case, in addition to the severely proclined upper anterior teeth and inadequate lip seal, which pose a high risk of trauma, the patient also presented with a constricted maxillary arch. Therefore, it was inadvisable to postpone treatment. A two-phase treatment plan was implemented promptly. The Phase I therapy aimed to retract the excessively proclined upper anterior teeth, expand the maxillary arch, coordinate the dental arch forms, and eliminate restrictions on mandibular growth.

Previous studies have shown that skeletal Class II malocclusion is frequently associated with a constricted maxillary arch [7,16]. A constricted maxillary arch can restrict the forward development of the mandible. Consequently, in patients with skeletal Class II malocclusion, the transverse coordination between the upper and lower dental arches should be carefully evaluated. Early arch expansion can be employed to eliminate the functional mandibular shift caused by the maxillary width discrepancy. Previous studies suggest that maxillary expansion in children with Class II mandibular retrognathia may contribute to mandibular growth. This also highlights the importance of early maxillary expansion therapy in children with Class II malocclusion accompanied by maxillary arch constriction [17]. The midpalatal suture begins to undergo synostosis around ages 14-15 in females and 15-16 in males. Therefore, conventional palatal expansion can effectively open the midpalatal suture when performed before puberty (generally before age 12) [18]. Arch width and cone beam computed tomography (CBCT)/linear measurements were recorded following protocols used in randomized trials comparing slow and rapid expansion [19]. Baseline three-dimensional arch dimensions were referenced to published CBCT norms for Class II Division 1 patients [20]. Compared to rapid maxillary expansion, slow maxillary expansion employs slower and lighter forces to open the midpalatal suture at a more gradual rate. This approach more closely mimics the physiological process, resulting in better tissue adaptation, reduced palatal tissue trauma, and greater long-term stability of the expansion outcomes [21].

In this case, the patient was 10 years old at the initial consultation. Cervical vertebral maturation analysis indicated a pre-pubertal growth stage. Therefore, slow maxillary expansion was employed to widen the constricted maxillary arch, concurrently with the use of a double-loop lip bumper to retract the excessively proclined upper anterior teeth. Previous studies have found that following maxillary expansion in patients with skeletal Class II malocclusion, the sagittal Class II discrepancy may self-correct [17]. During orthodontic treatment, radiographic monitoring of root and alveolar changes is recommended [22]. We also conducted radiographic evaluation upon completion of the patient's Phase I treatment. The results indicated that both the A-Nasion-B (ANB) angle and Wits appraisal showed a decreasing trend following Phase I maxillary expansion. The molar relationship also progressively improved from a full Class II to a Class I relationship. This phenomenon could be attributed to the fact that maxillary expansion eliminated the restrictive effect of the constricted maxillary arch on mandibular development, coupled with the patient's ongoing mandibular growth during the peak pubertal growth period. However, it is important to note that the improvement in cephalometric outcomes may also be attributed to potential errors in landmark identification and measurement [23], along with the patient's natural mandibular growth during the peak growth period. Moreover, two-dimensional cephalometric superimposition also presents inherent inaccuracies. At present, newer three-dimensional evaluation methods are available, enabling three-dimensional superimposition and volumetric comparison [24].

For skeletal Class II malocclusion with mandibular deficiency, treatment often involves mandibular advancement functional appliances during the peak pubertal growth period. This approach aims to stimulate mandibular growth and correct the sagittal discrepancy between the jaws [2,5]. However, traditional mandibular advancement functional appliance therapy inevitably carries risks of adverse effects, including mandibular clockwise rotation and labial inclination of the lower anterior teeth. Consequently, this treatment approach is contraindicated in patients with high-angle vertical patterns, vertical growth tendencies, or pre-existing labial proclination of the lower incisors [25]. In this case, the cephalometric results prior to Phase II treatment indicated an Frankfort-mandibular plane (FMA) angle of 32.1°. Since cephalometric reference values vary across different age groups, genders, and populations, and based on the reference values for the Chinese population [26,27], this value already suggested a tendency toward a high-angle pattern in the patient. However, the measured value of Sella-Gonion/Nasion-Menton (the ratio of posterior to anterior lower facial height) or S-Go/N-Me (%) remained within the normal range, indicating that the patient exhibited an average growth pattern. Therefore, considering that the patient still exhibited mandibular retrognathia, functional appliance therapy remained a preferred treatment option. However, in this case, the patient still presented with proclined lower anterior teeth after Phase I treatment, while the overjet had been reduced to 3 mm. The proclined lower incisors and the insufficient overjet could potentially limit mandibular advancement. Additionally, the bilateral molar relationship had partially self-adjusted. Consequently, we ultimately opted against functional appliance therapy and instead chose extraction-based orthodontics to retract both the upper and lower anterior teeth. Given the patient's peak growth period, Class II elastics were concurrently utilized to facilitate sagittal correction of the mandibular relationship [28]. However, the actual impact of functional appliances on Class II patients remains a subject of debate.

Numerous studies indicate that the primary treatment effects of functional devices are dentoalveolar in nature, with minimal skeletal changes [29]. The extraction of premolars for camouflage orthodontic represents another treatment option employing dental compensation in the correction of Class II malocclusion [30]. By removing two to four premolars, the Class II occlusal relationship can be corrected, accompanied by retraction of proclined anterior teeth and improvement of overbite and overjet. Decisions on extraction impact soft‑tissue outcomes and patient satisfaction [31]. Well-established ethnic variations in soft tissue characteristics exist among Asian and Caucasian populations. Overall, Asians present with more acute nasolabial angles, greater lip procumbence, and more convex facial profiles compared to Caucasian population, which explains the higher prevalence of tooth extraction in this demographic [32]. However, extraction and en-masse retraction protocols have been directly compared in Class II Division 1 cohorts, showing predictable dental compensation when skeletal change is limited [33]. Given the patient's convex facial profile and severely proclined upper anterior teeth present in this case, the decision was ultimately made to proceed with premolar extraction therapy during the Phase II treatment.

While the patient and his parents expressed considerable satisfaction with the treatment outcomes and the improvement in soft tissue profile, several aspects of this case merit consideration for further refinement. Firstly, during Phase I treatment, the upper anterior teeth were over-retracted, resulting in an excessively upright position that may have restricted forward mandibular growth. Furthermore, considering that the patient still presented with mandibular retrognathia after Phase I treatment, functional appliance therapy remained the preferred option. Accordingly, after achieving dental decompensation through extraction treatment, the adjunctive use of a fixed functional appliance combined with skeletal anchorage could potentially enhance mandibular advancement. Temporary skeletal anchorage can alter retraction mechanics and outcomes in Class II cases [34]. Additionally, long-term follow-up is required for this case to evaluate the stability of the treatment outcomes.

Thus, the limitations of this case include excessive retraction of the upper anterior teeth during Phase I treatment, which may have limited mandibular advancement, persistent mandibular retrognathia with dental compensation after treatment, as well as a lack of long-term follow-up.

## Conclusions

Angle Class II division 1 malocclusion is a type of malocclusion that significantly impacts the psychological health of adolescents. The key to treating Angle Class II division 1 malocclusion lies in targeting the peak pubertal growth period and fully utilizing the mandible's growth potential. For patients with Angle Class II division 1 malocclusion presenting with crowding and/or pronounced proclination, a Phase I functional appliance therapy is often recommended, typically followed by premolar extractions to alleviate crowding and/or improve the facial profile.

This case demonstrated that two-phase orthodontic treatment achieved favorable outcomes for Angle Class II Division 1 malocclusion, with significant improvement in the soft tissue profile. Despite certain limitations, the present case suggested that early orthodontic intervention should be considered for patients with Angle Class II Division 1 malocclusion accompanied by deep overbite causing occlusal trauma, proclined anterior teeth increasing the risk of dental injury, detrimental oral habits, or the presence of factors interfering with mandibular advancement, such as maxillary and maxillary dental arch constriction or individual anterior tooth lingual version.

## References

[1] Ghaffar F, Jan A, Akhtar O (2022). Comparative analysis of dentoskeletal changes of the twin block appliance and the Advansync2 appliance in treatment of skeletal class-II malocclusion in Pakistani population: a randomized clinical trial. Eur J Dent.

[2] Bimalrag BR, Ephraim R, Ayilliath A, Punathil S, James J, Venugopal J (2024). Cephalometric evaluation of the pre- and posttreatment changes after the correction of class II division 1 malocclusion with twin block appliance in mixed dentition. Int J Clin Pediatr Dent.

[3] Choi SH, Kim JS, Cha JY, Hwang CJ (2016). Effect of malocclusion severity on oral health-related quality of life and food intake ability in a Korean population. Am J Orthod Dentofacial Orthop.

[4] Lin M, Xie C, Yang H, Wu C, Ren A (2020). Prevalence of malocclusion in Chinese schoolchildren from 1991 to 2018: a systematic review and meta-analysis. Int J Paediatr Dent.

[5] Xu F, Fang Y, Sui X, Yao Y (2024). Comparison of twin block appliance and Herbst appliance in the treatment of Class II malocclusion among children: a meta-analysis. BMC Oral Health.

[6] Jaber ST, Hajeer MY, Burhan AS, Alam MK, Al-Ibrahim HM (2023). Treatment effectiveness of young adults using clear aligners versus buccal fixed appliances in class I malocclusion with first premolar extraction using the ABO-Objective Grading System: a randomized controlled clinical trial. Int Orthod.

[7] Fang B, Jin ZL, Bai YX (2021). Experts consensus on diagnostic and therapeutic strategies for malocclusions at early developing stage (Article in Chinese). Shanghai Kou Qiang Yi Xue.

[8] Idris G, Hajeer MY, Al-Jundi A (2019). Soft- and hard-tissue changes following treatment of Class II division 1 malocclusion with activator versus trainer: a randomized controlled trial. Eur J Orthod.

[9] Proffit WR (2006). The timing of early treatment: an overview. Am J Orthod Dentofacial Orthop.

[10] Lv Y, Yan B, Wang L (2012). Two-phase treatment of skeletal class II malocclusion with the combination of the twin-block appliance and high-pull headgear. Am J Orthod Dentofacial Orthop.

[11] George AM, Felicita AS, Milling Tania SD, Priyadharsini JV (2021). Systematic review on the genetic factors associated with skeletal class II malocclusion. Indian J Dent Res.

[12] McNamara JA (1981). Influence of respiratory pattern on craniofacial growth. Angle Orthod.

[13] Pereira TC, Furlan RM, Motta AR (2019). Relationship between mouth breathing etiology and maximum tongue pressure. Codas.

[14] Rasol OA, Hajeer MY, Sultan K, Ajaj MA, Burhan AS, Jaber ST, Aljabban O (2024). Evaluation of the best method for orthodontic correction of skeletal deep bites in growing patients: a systematic review. Cureus.

[15] Shaadouh RI, Hajeer MY, Awawdeh MA, Jaber ST, Mahmoud GA, Almasri IA (2024). Effectiveness of low-intensity electrical current in accelerating the en-masse retraction of the upper anterior teeth following first-premolar extraction in young adult patients with class II division 1 malocclusion: a randomized controlled clinical trial. Int Orthod.

[16] Fu K, Fang S, Fan X, Liu C, Zhang C, Liu J, Xiao D (2021). Analysis of dental and basal bone arch form correlations in skeletal class Ⅱ malocclusion. Am J Orthod Dentofacial Orthop.

[17] Kotarska M, Kucukkeles N, Lis J, Kawala B, Rumin K, Sarul M (2022). Changes in the mandible following rapid maxillary expansion in children with class II malocclusion: a systematic review. Diagnostics (Basel).

[18] Ciambotti C, Ngan P, Durkee M, Kohli K, Kim H (2001). A comparison of dental and dentoalveolar changes between rapid palatal expansion and nickel-titanium palatal expansion appliances. Am J Orthod Dentofacial Orthop.

[19] Rabah N, Al-Ibrahim HM, Hajeer MY, Ajaj MA (2022). Evaluation of rapid versus slow maxillary expansion in early adolescent patients with skeletal maxillary constriction using cone-beam computed tomography: a short-term follow-up randomized controlled trial. Dent Med Probl.

[20] Al-Hilal LH, Sultan K, Hajeer MY, Mahmoud G, Wanli AA (2018). An evaluation of mandibular dental and basal arch dimensions in class I and class II division 1 adult Syrian patients using cone-beam computed tomography. J Contemp Dent Pract.

[21] Agarwal A, Mathur R (2010). Maxillary expansion. Int J Clin Pediatr Dent.

[22] Kara-Boulad JM, Burhan AS, Hajeer MY, Nawaya FR, Jaber ST (2025). CBCT-based assessment of apical root resorption and alveolar bone height following orthodontic treatment of class I moderate crowding with labial vs. lingual fixed appliances in young adults: a randomized controlled trial. Int Orthod.

[23] Prasad S, Denotti G, Farella M (2022). Effect of prior knowledge about treatment on cephalometric measurements. J Orthod.

[24] Hajeer MY, Mao Z, Millett DT, Ayoub AF, Siebert JP (2005). A new three-dimensional method of assessing facial volumetric changes after orthognathic treatment. Cleft Palate Craniofac J.

[25] Brito DB, Bellini-Pereira SA, Fonçatti CF, Henriques JF, Janson G (2023). Treatment effects of the MARA appliance and activator-headgear combined with fixed appliances in class II division 1 malocclusion patients: a retrospective longitudinal study. Dental Press J Orthod.

[26] Wu S, Wang T, Kang X, Wang X, Jiao Y, Du X, Zou R (2023). Hyoid bone position in subjects with different facial growth patterns of different dental ages. Cranio.

[27] Xiao D, Gao H, Ren Y (2011). Craniofacial morphological characteristics of Chinese adults with normal occlusion and different skeletal divergence. Eur J Orthod.

[28] Dianiskova S, Rongo R, Buono R, Franchi L, Michelotti A, D'Antò V (2022). Treatment of mild class II malocclusion in growing patients with clear aligners versus fixed multibracket therapy: a retrospective study. Orthod Craniofac Res.

[29] Zymperdikas VF, Koretsi V, Papageorgiou SN, Papadopoulos MA (2016). Treatment effects of fixed functional appliances in patients with Class II malocclusion: a systematic review and meta-analysis. Eur J Orthod.

[30] George SM, Campbell PM, Tadlock LP, Schneiderman E, Buschang PH (2021). Keys to class II correction: a comparison of 2 extraction protocols. Am J Orthod Dentofacial Orthop.

[31] Alhafi ZM, Hajeer MY, Latifeh Y (2024). The impact of non-extraction orthodontic treatment on the oral-health-related quality of life between a modified aligner appliance with NiTi springs and the traditional fixed appliances: a randomized controlled clinical trial. Medicina (Kaunas).

[32] Gu Y, McNamara JA Jr, Sigler LM, Baccetti T (2011). Comparison of craniofacial characteristics of typical Chinese and Caucasian young adults. Eur J Orthod.

[33] Khlef HN, Hajeer MY, Ajaj MA, Heshmeh O, Youssef N, Mahaini L (2020). The effectiveness of traditional corticotomy vs flapless corticotomy in miniscrew-supported en-masse retraction of maxillary anterior teeth in patients with class II division 1 malocclusion: a single-centered, randomized controlled clinical trial. Am J Orthod Dentofacial Orthop.

[34] Al-Sibaie S, Hajeer MY (2014). Assessment of changes following en-masse retraction with mini-implants anchorage compared to two-step retraction with conventional anchorage in patients with class II division 1 malocclusion: a randomized controlled trial. Eur J Orthod.

